# Paramagnetic rim lesions are associated with choroid plexus inflammation and expansion over 5 years in people with multiple sclerosis

**DOI:** 10.1007/s00415-025-13309-4

**Published:** 2025-08-21

**Authors:** Jack A. Reeves, Ashley Tranquille, Alexander Bartnik, Maryam Mohebbi, Fahad Salman, Dejan Jakimovski, Ferdinand Schweser, Bianca Weinstock-Guttman, Michael G. Dwyer, Eleonora Tavazzi, Robert Zivadinov, Niels Bergsland

**Affiliations:** 1 Department of Neurology, Buffalo Neuroimaging Analysis Center, Jacobs School of Medicine and Biomedical Sciences, University at Buffalo, State University of New York, 77 Goodell Street, Suite 440, Buffalo, NY 14203 USA; 2 Department of Imaging Sciences, Strong Memorial Hospital, University of Rochester, Rochester, NY USA; 3 Center for Biomedical Imaging at the Clinical Translational Science Institute, University at Buffalo, State University of New York, Buffalo, NY USA; 4 Jacobs Neurological Institute, Buffalo, NY USA; 5 Multiple Sclerosis Centre, IRCCS Mondino Foundation, Pavia, Italy

**Keywords:** Choroid plexus, Chronic active lesion, Iron rim lesion, Multiple sclerosis, Paramagnetic rim lesion

## Abstract

**Background:**

Chronic active white matter inflammation is linked with multiple sclerosis (MS) clinical severity and is likely integral in MS progression. It has also been associated with choroid plexus (CP) inflammation in vivo, pointing to a potential pathophysiological link between the two phenomena. However, how these aspects of the disease co-evolve over time remains poorly understood nor has their relationship been specifically assessed in people with progressive MS (pwPMS).

**Methods:**

In this retrospective analysis of a longitudinal study, 129 people with MS (pwMS; 86 people with relapsing remitting (pwRRMS) and 43 pwPMS) were imaged with 3 T MRI at baseline and after 5.4 years of follow-up. CP volume and CP pseudo-T2 (pT2) were calculated as measures reflecting CP inflammation in MS. Paramagnetic rim lesions (PRLs), a marker of chronic active white matter inflammation, were assessed at baseline for the whole cohort, and longitudinally in 96 pwMS with available data (66 pwRRMS, 32 pwPMS). Baseline and longitudinal associations between the CP and PRLs, including interactions with disease course, were assessed, adjusted for covariates including age and sex.

**Results:**

PwMS with PRLs had significantly larger CP volume at baseline than pwMS without PRLs after correcting for age, sex, and disease duration (mean difference: 0.3 ± 1.2 mL; *p* = 0.042). Longitudinally, baseline PRL number was associated with increased CP volume in pwPMS (*B* = 0.22 mL/mL; *p* < 0.001), but not people with relapsing–remitting MS (*B* = − 0.01 mL/mL; *p* > 0.7).

**Conclusion:**

Increased CP volume is related to chronic white matter inflammation, particularly in pwPMS.

## Introduction

Multiple sclerosis (MS) is an inflammatory immune-mediated disease characterized by injury to the gray matter (GM) and white matter (WM) of the central nervous system (CNS) [1, 2]. In MS, it is widely recognized that peripheral immune cells invade the CNS via an impaired blood–brain barrier (BBB) [2]. However, the role of the blood-cerebrospinal fluid barrier (BCSFB) in immune cell trafficking into the CNS has come under increased scrutiny over recent years as well [3–5]. The choroid plexus (CP), which makes up the BCSFB along with the arachnoid mater, is a vascular structure comprised of epithelial cells found in the lateral, third and fourth ventricles of the brain [6]. Interactions between CSF and blood permits the migration of humoral immune cells through tight junctions of the CP [6, 7].

A number of histopathological studies have found that the CP in people with MS (pwMS) is characterized by several changes, including the selective loss of tight junction protein claudin-3 [8], accumulation of granulocytes and *T* cells, [9] intense human leukocyte antigen-DR staining, [10] and substantial inflammation [11]. Recent in vivo imaging studies have also highlighted the involvement of the CP in pwMS [12]. The most widely reported finding has been increased CP volume compared to healthy controls [13–16]. While it is difficult to tie its enlargement to a specific biological process, the phenomenon is related to, at least in part, CP inflammation, as evidenced by both PET and MRI-derived measures [15, 16].

Initial studies suggested that CP enlargement is related to acute inflammatory activity throughout the brain, as reflected by increased relapse rates, presence of gadolinium-enhancing lesions, and future disease activity over the relatively short term [3, 16]. Recently, associations with chronic neuroinflammation have also been investigated. Specifically, it has been shown that increased CP volume relates to chronic active lesions in the WM, as reflected by both paramagnetic rim lesions (PRL) [17] and expansion of chronic lesions [13, 14, 18]. However, these studies were either limited to people with relapsing–remitting MS (pwRRMS) or combined pwRRMS and people with progressive MS (pwPMS) into a single group. As such, the relationship between CP inflammation and chronic WM inflammation in pwPMS is less clear.

In this study, we used 3 T MRI to analyze cross-sectional and longitudinal associations between CP inflammation and chronic WM inflammation in a cohort consisting of pwRRMS and pwPMS. In particular, we assessed the relationship between CP inflammation and PRLs at baseline and their associations with respect to changes over the follow-up, while also considering the influence of disease course on these outcomes.

## Materials methods

### Study population

Participants were from a previous prospective longitudinal study, which investigated cardiovascular, environmental, and genetic factors in multiple sclerosis (CEG-MS) [19]. The CEG-MS study’s inclusion criteria included participants who were between 18 and 75 years of age and met the 2010-revision of the McDonald criteria for the diagnosis of MS. CEG-MS exclusion criteria included contraindications for MRI examination, pregnancy or nursing, and clinical relapse or administration of intravenous corticosteroid therapy within 30 days of the MRI examination. Comprehensive information regarding the CEG-MS inclusion and exclusion criteria can be found elsewhere [19]. Approval for the CEG-MS study was obtained from the Institutional Review Board of the University at Buffalo, and all participants provided written informed consent. In accordance with the Declaration of Helsinki. Additional inclusion criteria for the present study included availability of MRI (including a 3D T2*-weighted gradient echo for QSM processing) and clinical assessment baseline MRI scan within 30 days of each other. All eligible participants were included in baseline-only comparisons as part of the “Full Cohort”, and the subset of participants who had 5 years MRI and clinical assessments available were included in longitudinal analyses as part of a “Longitudinal PRL Cohort”. Participants were excluded from the current study if did not meet all of the aforementioned criteria or artifacts on baseline MRI prevented analysis.

### Image acquisition

Imaging was performed on a 3 T scanner (Signa Excite HD 12.0; GE HealthCare, Milwaukee, WI, USA) using an eight-channel head-and-neck coil and used an axial three-dimensional gradient-echo sequence with first-order flow compensation in read and slice directions (matrix, 512 × 192 × 64; 0.5 × 1 × 2 mm^3^; 12°flip; echo time (TE) = 22 ms; repetition time (TR) = 40 ms; bandwidth, 13.89 kHz). At the time when our study began collecting data in 2009, only a single echo gradient echo sequence was available on our scanner. The following additional sequences were acquired axially during the same imaging session for all subjects with 1 × 1 × 3 mm^3^ voxel sizes, without gap: spin-echo T1-weighted (T1w) imaging (matrix, 256 mm × 192 mm; FOV, 256 mm × 192 mm; TE = 16 ms; TR = 600 ms); FLAIR (matrix, 256 mm × 192 mm; FOV, 256 mm × 192 mm; TE = 120 ms; inversion time (TI) = 2100 ms; TR = 8500 ms; flip angle = 90°; echo-train length, 24); dual fast spin-echo proton density (PD)- and T2-weighted imaging (matrix, 256 mm × 192 mm; FOV, 256 mm × 192 mm; TE1 = 9 ms; TE2 = 98 ms; TR = 5300 ms; echo-train length = 14). In addition, an axial 3D high-resolution T1w inversion recovery fast spoiled-gradient echo (IR-FSPGR) was acquired (TE = 2.8 ms; TI = 900 ms; TR = 5.9 ms; flip angle, 10°; isotropic 1 mm resolution).

### Image analysis

T2 lesions were quantified at baseline using a semi-automated contouring technique, as previously reported using Java Image Manipulation (JIM, version 6.0) [20]. Lesions were drawn on the FLAIR image while rigidly aligned PD/T2 weighted images were simultaneously reviewed to improve confidence in the segmentation. Follow-up FLAIR and PD/T2 weighted images were rigidly aligned into the same space as the baseline FLAIR and segmentations were adjusted to account for changes in lesion size. Counting of new or enlarging lesions was not performed. The QSM reconstruction methodology has been thoroughly explained elsewhere [21]. QSM images were referenced to the whole brain.

**Fig. 1:**
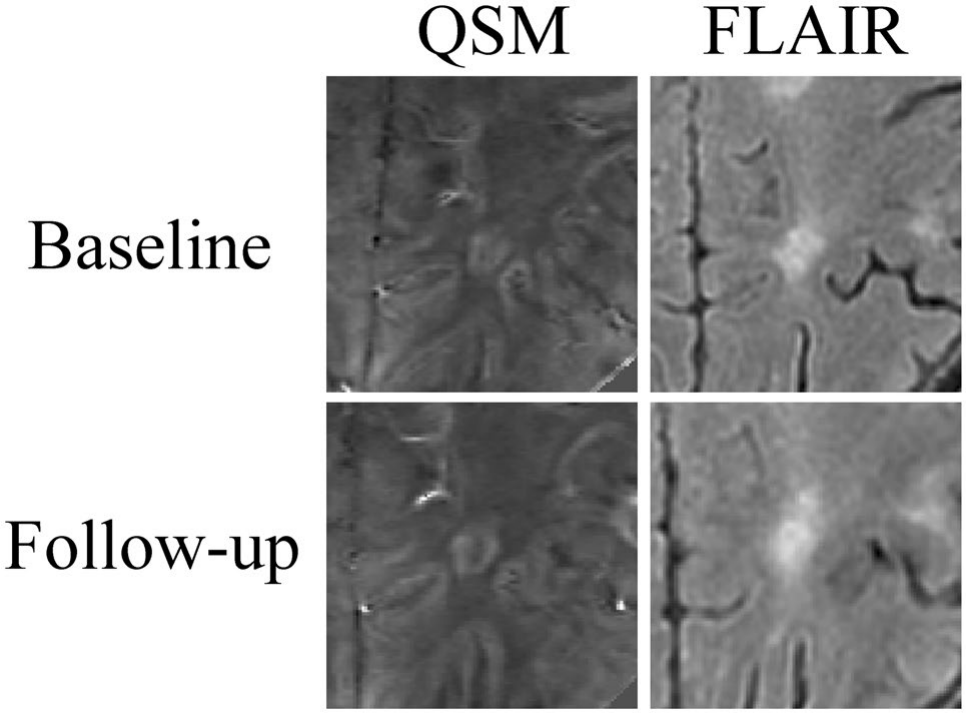
Representative baseline and follow-up fluid-attenuated inversion recovery (FLAIR) and quantitative susceptibility mapping (QSM) images showing a persisting paramagnetic rim lesion. QSM images are windowed from − 0.1 to 0.2 ppb

FLAIR images were rigidly aligned to the magnitude image of the sequence used to derive QSM images and T2 lesions were then brought into native QSM space. A researcher with 3 years of lesion classification experience (J.R.) identified and semi-automatically segmented PRLs on baseline QSM images using JIM software while simultaneously reviewing the FLAIR image and corresponding T2 lesion mask. Subsequently, senior researchers with over 10 years of lesion classification experience reviewed the QSM images (R.Z., M.D., and N.B.) and PRL classifications until achieving group consensus. Baseline and follow-up QSMs were then linearly co-registered, and accuracy was assessed by a researcher with 8 years of neuroimaging experience (A.B.). PRLs were then reconciled between baseline and follow-up and classified as persisting (rim present at baseline and follow-up), newly appearing (rim present only at follow-up), or disappearing (rim present at baseline but not follow-up). An example of a persisting PRL is shown in Fig. 1. The rater and consensus review group were blinded to clinical and demographic information for all classifications. Further information on rater training and reliability analyses can be found elsewhere [22]. The PRL criteria followed the 2024 NAIMS consensus statement on imaging chronic active lesions [23]. Namely, PRLs encompassed the presence of a paramagnetic rim contiguous with at least 2/3 of the outer lesion edge, had a diamagnetic core relative to surrounding extra-lesional white matter, had a maximum diameter of ≥ 3 mm, and exhibited non-enhancement on post-contrast T1 sequence [23].

Normalized lateral ventricular volume at baseline was obtained from lesion-filled [24] 3D T1w images using SIENAX [25]. Segmentation and volumetry of the CP are described in detail elsewhere [15]. In summary, initial CP segmentations were obtained via FreeSurfer’s recon-all pipeline, [26] which were further refined using a previously described Gaussian Mixture Model method [27]. The resulting segmentations were brought into native 3D T1w space and manually corrected by reviewers (N.B., E.T.) blinded to clinical data. The resulting volumes were normalized for head size using the SIENAX-derived scaling factor.

Pseudo T2 (pT2) maps were generated from the dual-echo SE sequence, as previously described [15]. Briefly, the maps are calculated on a voxel-wise basis by taking the difference between the two TEs and dividing by the log of the ratio between the signal intensities in the first and second echo. pT2 increases with water content and is thought to reflect, at least in part, inflammation [28]. The PD image was rigidly aligned to the native 3D T1w image and the resulting matrix was used to bring along the pT2 map while using spline interpolation. All registrations were performed using FSL’s Linear Image Registration Tool (FLIRT) and visually checked to confirm accurate alignment.

### Statistical analyses

#### Univariate comparisons

Analysis of covariance (ANCOVA) models corrected for baseline age, sex, and baseline disease duration were used to compare CP measures between pwMS with at least one PRL at baseline (PRL+) and pwMS without any PRLs (PRL-). CP volume and pT2 were compared between the groups at baseline and for 5 years longitudinal changes. *P* values lower than 0.05 were considered statistically significant. SPSS version 29.0 (IBM, Armonk, NY, United States) was used for all statistical analysis.

#### Model-based associations between baseline PRLs and baseline and longitudinal choroid plexus measurements

We used a stepwise approach to evaluate the relationship between PRLs and CP. Our initial analysis assessed whether baseline PRL number and volume were associated with baseline CP measures (volume and pT2). We then assessed whether baseline PRL number and volume predicted dynamic longitudinal changes in CP measures (change in CP volume and change in CP pT2). For each analysis, initial models evaluated whether any of these potential associations were disease course-dependent by the inclusion of PRL by disease course interaction terms (either baseline PRL number by baseline disease course or baseline PRL volume by baseline disease course). If a significant interaction effect was found, the interaction effect was removed from the model and RRMS and PMS groups were analyzed separately for main effect of either baseline PRL number or baseline PRL volume. If no interaction effect was found, the interaction effect was removed and the whole cohort was analyzed for the main effect of either baseline PRL number or baseline PRL volume. All models included covariates of sex, baseline age, baseline disease duration, baseline T2-LV, baseline EDSS, and baseline normalized lateral ventricular volume, and the final whole-cohort models of PRL main effect included disease course as a covariate. Models of 5 years CP changes also included covariates of follow-up time and baseline CP pT2 (for change in CP pT2 models) or baseline CP volume (for change in CP volume models).

Following these analyses, we assessed whether dynamic changes in CP were related to changes in PRLs. Linear regression models compared predictor variables of change in CP volume and change in CP pT2 to outcome variables of change in PRL number or change in PRL volume. CP change by disease course interaction terms were tested and subgroup analyses were conducted for significant terms. Models controlled for sex, baseline age, baseline disease duration, baseline T2-LV, baseline EDSS, time to follow-up, baseline normalized lateral ventricular volume, and baseline PRL number (for change in PRL number and new PRL appearance models) or volume (for change in PRL volume models), and the final whole-cohort models of PRL main effect included disease course as a covariate.

Model assumptions were assessed using standard diagnostic plots. *P* values lower than 0.05 were considered statistically significant.

#### Descriptive analysis of conversion to secondary progressive MS

Although we were underpowered to statistically compare pwRRMS at baseline that converted to a secondary progressive phenotype, we explored CP and PRL features compared to those that did not as well as those who already had progressive MS.

## Results

### Demographics and clinical characteristics

The Full Cohort included 129 pwMS. Of these, 98 had follow-up PRL data and were included in the Longitudinal PRL Cohort. Full demographic and clinical characteristics for both cohorts are shown in Table 1.

**Table 1:** Demographic characteristics

	Full Cohort			Longitudinal PRL Cohort		
	Baseline	Follow-up	Change	Baseline	Follow-up	Change
N	129	129	129	98	98	98
Disease course	86 pwRRMS, 43 pwPMS (36 pwSPMS, 7 pwPPMS)	79 pwRRMS, 50 pwPMS (43 pwSPMS, 7 pwPPMS)	–	66 pwRRMS, 32 pwPMS (25 pwSPMS, 7 pwPMS)	61 pwRRMS, 37 pwPMS (30 pwSPMS, 7 pwPPMS)	–
Age, mean ± S.D	47.0 ± 10.8 years	53.0 ± 10.9 years	–	47.3 ± 11.0 years	53.2 ± 11.1 years	–
Sex	96 female, 33 male	96 female, 33 male	–	75 female, 23 male	75 female, 23 male	–
Disease duration, years	14.8 ± 9.8 years	20.5 ± 9.9 years	–	14.8 ± 9.6 years	20.4 ± 9.7 years	–
Time to follow-up	–	5.4 ± 0.6 years	–	–	5.3 ± 0.5 years	–
EDSS, median [IQR]	2.5 [1.5 − 5.3]	3.5 [2.0 − 6.0]	**Mean ± S.D. 0.4 ± 1.0 *p* < 0.001**^**a**^	2.75 [1.5 − 5.1]	3.5 [2.0 − 6.0]	**Mean ± S.D. 0.3 ± 1.0 *p* = 0.006**^**a**^
T2 LV, mean ± S.D	16.3 ± 20.8 mL	16.6 ± 19.8 mL	0.4 ± 5.3 mL *p* = 0.418^a^	16.9 ± 22.2 mL	17.5 ± 21.3 mL	0.6 ± 5.5 mL *p* = 0.245^a^
PRL presence, % (n)	50.4% (65)	–	–	49.0% (48)	40.0% (39)	− 9.0% *p* = 0.196^b^
PRL number, mean ± S.D	2.0 ± 3.5	–	–	1.8 ± 3.0	1.4 ± 2.8	** − 0.4 ± 1.5 *p* = 0.007**^**a**^
PRL volume, mean ± S.D	1.1 ± 2.4 mL	–	–	1.0 ± 1.9 mL	0.9 ± 1.9 mL	**−0.1 ± 0.4 mL *p* = 0.005**^**a**^
Newly appearing PRL incidence, % (n)	–	–	–	–	14.3% (14)	–
Normalized CP volume, mean ± S.D	2.6 ± 0.8 mL	2.7 ± 0.9 mL	0.1 ± 0.7 mL *p* = 0.251^a^	2.6 ± 0.9 mL	2.7 ± 0.9 mL	0.1 ± 0.7 mL *p* = 0.326^a^
CP pT2 value, mean ± S.D	1.0 ± 0.5 s	1.2 ± 0.5 s	**0.1 ± 0.5 s *p* = 0.019**^**a**^	1.1 ± 0.5 s	1.2 ± 0.5 s	**0.1 ± 0.5 s *p* = 0.040**^**a**^

*P* values < 0.05 are bolded

EDSS-Expanded Disability Status Scale, IQR-interquartile range, PRL-paramagnetic rim lesion, pT2-pseudo-T2, pwRRMS-people with relapsing-remitting multiple sclerosis, pwPMS-people with progressive multiple sclerosis, pwPPMS-people with primary progressive multiple sclerosis, pwSPMS-people with secondary progressive multiple sclerosis, S.D.-standard deviation, T2-LV-T2 lesion volume; CP-choroid plexus

^a^One-sample, 2-tailed *t*-test, ^b^Chi-squared test

At baseline, the Full Cohort consisted of 86 pwRRMS and 43 pwPMS (36 people with secondary progressive MS (pwSPMS), 7 people with primary progressive MS (pwPPMS)), with 96 females and 33 males. They had a mean age of 47.0 ± 10.8 years, mean disease duration 14.8 ± 9.8 years, median EDSS 2.5 (1.5 − 5.3 IQR), mean T2-LV 16.3 ± 20.8 mL, mean normalized CP volume of 2.6 ± 0.8 mL, and mean choroid plexus pT2 of 1.0 ± 0.5 s. Of these, 50.4% (65/129) had at least one PRL, with an overall mean PRL number of 2.0 ± 3.5 and mean PRL volume of 1.1 ± 2.4 mL. The Longitudinal PRL Cohort consisted of 66 pwRRMS and 32 pwPMS (25 pwSPMS, 7 pwPMS) at baseline, with 75 females and 23 males. This cohort was comparable to the Full Cohort in terms of baseline clinical, demographic, lesion, and CP characteristics (results not shown).

The mean follow-up time for the Full Cohort was 5.4 ± 0.6 years. Over follow-up, the Full Cohort increased in EDSS by a mean of 0.4 ± 1 (*p* < 0.001) and increased in CP pT2 by 0.1 ± 0.5 s (*p* = 0.019), but had similar T2-LV and normalized CP volume to baseline (*p* > 0.2). The Longitudinal PRL Cohort had similar increases in EDSS and CP pT2 (*p* < 0.04), and no increase in T2-LV or normalized CP volume (*p* > 0.2). PRL number (−0.4 ± 1.5, *p* = 0.007) and volume (−0.1 ± 0.4 mL, *p* = 0.005) decreased at follow-up, but PRL prevalence was similar (*p* > 0.15).

### Choroid plexus comparisons between PRL + and PRL − pwMS

Table 2 shows baseline cross-sectional and 5 years longitudinal CP comparisons between the PRL + and PRL − pwMS in the Full Cohort, corrected for baseline age, sex, and baseline disease duration. The baseline CP volume was greater in PRL + pwMS as compared to PRL- pwMS (uncorrected means: 2.7 ± 0.8 mL for PRL + and 2.5 ± 0.9 mL for PRL-; model-based *p* = 0.042). Baseline CP pT2 trended higher in the PRL + group but did not reach significance (uncorrected means: 1.2 ± 0.5 s for PRL + and 1.0 ± 0.5 s for PRL−; model-based *p* = 0.066). PRL + and PRL- groups did not differ in 5 years change in CP volume (*p* = 0.151) or change in CP pT2 (*p* = 0.354).

**Table 2:** Choroid plexus comparisons between PRL + and PRL − pwMS in the Full Cohort

	PRL + (*n* = 65)	PRL − (*n* = 64)	*p* value^a^
Baseline	–	–	–
Normalized choroid volume	2.7 ± 0.8 mL	2.5 ± 0.9 mL	***p* = 0.042**
Choroid plexus pT2	1.2 ± 0.5 s	1.0 ± 0.5 s	*p* = 0.066
5 years change	–	–	–
Normalized choroid plexus volume	0.2 ± 0.7 mL	− 1.6E − 3 ± 0.7 mL	*p* = 0.151
Choroid plexus pT2	0.1 ± 0.5 s	0.1 ± 0.4 s	*p* = 0.354

mL-milliliters, pT2-pseudo-T2, PRL-paramagnetic rim lesion.

ANCOVAs with covariates of baseline age, sex, and baseline disease duration were used for all comparison. Uncorrected CP values are shown with model-based *p* values. *P* values < 0.05 are bolded

### Model-based evaluation of associations between choroid plexus measurements and baseline PRLs in the Full Cohort

Table 3 shows model-based associations between baseline PRLs and baseline and longitudinal choroid plexus measurements. The 5 years change in CP volume had significant interaction effects of baseline disease course by baseline PRL volume and baseline disease course by baseline PRL number (*p* = 0.001 for both). In subgroup analyses, only the pwPMS group showed a significant association between change in CP volume and baseline PRL volume (*B* = 0.22 mL CP increase per mL PRL, *p* < 0.001) and baseline PRL number (*B* = 0.15 mL CP increase per PRL, *p* < 0.001).

**Table 3:** Model-based associations between baseline PRLs and baseline and longitudinal choroid plexus measurements in the Baseline PRL Cohort

	Baseline CP pT2	Baseline CP Volume	Change in CP pT2	Change in CP volume
Baseline PRL volume	Interaction *p* = 0.900 Whole cohort *B* = 8.4E − 3 mL/mL *p* = 0.676	Interaction *p* = 0.625 Whole cohort *B* = 0.04 mL/mL *p* = 0.273	**Interaction *p* = 0.015** pwRRMS: *B* = 0.02 s/mL *p* = 0.359 pwPMS: *B* = − 0.02 s/mL *p* = 0.374	**Interaction *p* < 0.001** pwRRMS: B = − 0.01 mL/mL *p* = 0.715 **pwPMS: *B* = 0.22 mL/mL *p* < 0.001**
Baseline PRL number	Interaction *p* = 0.489 Whole cohort B = 9.4E − 3 mL/PRL *p* = 0.486	Interaction *p* = 0.399 Whole cohort *B* = 0.02 mL/PRL *p* = 0.203	**Interaction *p* = 0.010** pwRRMS: *B* = 0.02 s/PRL *p* = 0.126 pwPMS: *B* = − 7.2E − 3 s/PRL *p* = 0.697	**Interaction *p* = 0.002** pwRRMS: *B* = − 6E − 3 mL/PRL *p* = 0.771 **pwPMS: *B* = 0.15 mL/PRL *p* < 0.001**

“Baseline CP” models included covariates of sex, baseline age, baseline disease duration, baseline EDSS, baseline disease course, baseline T2-LV, and normalized lateral ventricular volume. “Change in CP” models also include covariates of baseline CP value and time to follow-up. Interaction terms were removed from the models for estimation of main effects. For main effects, beta values are given with associated *p* values in parentheses

Neither baseline PRL volume nor baseline PRL number was associated with change in CP pT2 (*p* > 0.15) or change in CP volume (*p* > 0.2). 5 years change in CP pT2 had significant interaction effects of baseline disease course by baseline PRL volume and baseline disease course by baseline PRL number (*p* < 0.024). However, neither baseline PRL volume nor baseline PRL number were significantly associated with 5 years change in CP pT2 when analyzing the pwRRMS and pwPMS groups separately (*p* > 0.15).

### Model-based associations between longitudinal PRL changes and longitudinal choroid plexus changes.

Table 4 shows the associations between 5 years changes in PRL volume and PRL number and 5 years changes in CP volume and pT2 in the Longitudinal PRL Cohort. No CP change by disease course terms were significant (*p* > 0.1), and all main effects were non-significant (*p* > 0.2).

**Table 4:** Model-based associations between longitudinal PRL changes and longitudinal choroid plexus changes in the Longitudinal PRL Cohort

	Change in PRL volume	Change in PRL number
Change in CP volume	*B* = 0.14 mL/mL (*p* = 0.335)	*B* = 0.03 PRL/mL (*p* = 0.509)
Change in CP pT2	*B* = − 0.07 mL/sec (*p* = 0.486)	*B* = − 0.01 PRL/mL (*p* = 0.759)

CP-choroid plexus; mL-milliliters, pT2-pseudo-T2, PRL-paramagnetic rim lesion.

Models included covariates of sex, baseline age, baseline disease duration, baseline EDSS, baseline disease course, baseline T2-LV, change in PRL volume or number, time to follow-up, and baseline normalized lateral ventricular volume. B values are given with associated *p* values in parentheses

### Descriptive analysis of conversion to progressive MS

Baseline CP volumetric measures as well as changes over follow-up in PwRRMS that had a progressive phenotype at follow-up were more similar to those that already had a progressive phenotype at baseline. Specifically, mean baseline CP volume was 2.7 mL while volume change was 0.3 mL in converters compared to 2.5 mL and 0.01 mL in stable RRMS versus 2.7 mL and 0.2 mL in progressive MS at baseline. However, baseline CP pT2 was more similar between converters and stable RRMS (1.1 s vs. 1.0 s) compared to PMS (1.3 s) while converters had the greatest longitudinal increase (0.2 s) compared to stable RRMS (0.1 s) and PMS (< 0.1 s).

Mean baseline PRL number was also greatest in converters (4.8) compared to 1.7 and 2.0 in stable RRMS and PMS, respectively. Similarly, mean baseline PRL volume was greatest in converters (2.3 mL) compared to 1.0 mL and 1.2 mL in stable RRMS and PMS, respectively. In terms of PRL changes over the follow-up, all groups had a similar mean number of new PRLs (0.1) while the greatest decrease in volume was seen in the converters (−0.3 mL) compared to − 0.1 mL and − 0.2 mL in stable RRMS and PMS, respectively.

## Discussion

In this study, we compared CP volume and PRLs at baseline and longitudinally over 5 years in a fairly large cohort of pwMS. We reproduced previous results that baseline CP volume is associated with baseline PRL presence (yes/no) and PRL volume, [17] while also showing the relationship in pwPMS as well. We then extended these results by showing that baseline PRLs predict longitudinal increases in CP volumes, but only in pwPMS. These results point to a strong link, and potential pathophysiological overlap, between chronic active inflammation in the brain parenchyma and an inflamed choroid plexus.

Our main finding was that baseline PRL load (number and volume) was associated with greater future changes in CP volume, but only in the pwPMS group. Several studies have shown that while CP volume increases very early in the disease, [29, 30] CP volume is similar between pwRRMS and pwPMS [15, 17]. Our results shed light on these findings by showing that CP expansion later in the disease may per particularly relevant in a subset of pwPMS, namely those with elevated levels of ongoing chronic active inflammation. This suggests that with respect to pwPMS, PRLs relate to the observed increase in CP volume, possibly due to the maintenance of a proinflammatory milieu throughout the CNS. Activated microglia, which are present at the rims of PRLs, have been shown to release proinflammatory cytokines [31, 32]. Moreover, a study of healthy individuals found that aging was associated with impaired CP functioning and altered structure, as reflected by decreased cerebral blood flow and increased mean diffusivity and volume, respectively [33]. It is thus tempting to speculate that activated microglia-induced inflammation and aging further aggravate the already impaired CP in MS with respect to its role in regulating immune cell trafficking. Furthermore, our findings suggest that disruption of barrier sites, at least with respect to the BCSB, remains relevant in progressive MS and that inflammation is not entirely compartmentalized behind intact barriers. Our descriptive comparisons between those pwRRMS that converted to a progressive phenotype over the follow-up also support the relevance of chronic inflammation in both the CP and the WM in leading to disability, as previously shown [15, 34].

Our findings draw attention to the role of CSF, which is characterized by numerous pathological changes in pwMS, [35] in linking CP inflammation and PRLs. Proinflammatory as well as cytotoxic molecules, which stem at least in part from the CP, [4] likely diffuse through the CSF and cross the ependymal cells that separate the ventricular system from the CNS. A number of recent studies support this hypothesis. For example, CP enlargement in pwMS has been shown to be associated with decreased probability of successful remyelination [36]. However, while PRLs are thought to be more destructive in nature than non-PRLs, [37] there does not appear to be a difference in their prevalence with respect to their distances from ventricular CSF [38]. While the exact mechanisms that determine the long-term fate of a lesion with respect to becoming a PRL remain poorly understood, our results suggest that CP inflammation and PRL dynamics are intertwined in pwPMS.

As of now, there are a limited number of studies that have assessed CSF profiles and PRLs in pwMS. However, one recent study found that a pathologically-elevated albumin quotient was present exclusively in pwMS with at least one PRL [39]. The authors interpreted the elevated albumin quotient in the context of increased BBB permeability/disruption, as is typically reported in the literature [40]. However, an alternative, or at least complementary, explanation is that pathologically-elevated albumin levels may in part stem from the CP. While albumin crosses the BCSFB in normal physiological conditions, [41] it is conceivable that an impaired CP in pwMS may facilitate excessive transfer from the blood into the CSF. As of now, the relationship between CSF markers and CP changes in pwMS are largely missing from the literature. As such, longitudinal studies that include serial CSF assessments and imaging protocols that facilitate the quantification of CP inflammation and PRLs may help shed further light on how chronic inflammation evolves throughout the disease.

There are several limitations to the current study. First, we acknowledge that we only assessed the CP within the lateral ventricles. Additionally, our technique for mapping T2 times used a sequence with 3 mm thick slices while a 1 mm isotropic acquisition was used for CP segmentation. Thus, there is increased risk of partial volume contamination when sampling the pT2 map into the 3D T1 space. However, it is important to consider that partial volume effects should scale inversely with CP volume. As such, we do not believe that partial voluming had a substantial impact on our results. Nevertheless, future studies would benefit from a higher quality T2 mapping acquisition, both in terms of voxel size and number of echoes. Moreover, we do not have CSF data from the pwMS included in our study, the availability of which would have potentially allowed us to draw firmer conclusions. Finally, we did not study healthy controls in the current study as our focus was specifically on PRLs, which are not expected to be found in such individuals. In an earlier study investigating CP volume and CP pT2, we found that both were significantly increased at baseline in pwMS compared to healthy individuals. Over the course of 5 years, CP volume increased at a similar rate while a greater increase in CP pT2 was evidenced in the healthy individuals [15]. In light of these earlier findings, it is difficult to unequivocally interpret the association between changes in the CP and PRLs and additional studies are warranted.

## Conclusion

Our findings support a relationship between chronic white matter inflammation, as reflected by PRLs, and CP inflammation, particularly in the progressive forms of MS. Future studies should explore the mechanistic link between these phenomena, which may further elucidate the evolution of MS pathophysiology throughout the disease.
